# Subjective memory measures: Metamemory questionnaires currently in use

**DOI:** 10.1177/17470218231183855

**Published:** 2023-07-03

**Authors:** Yashoda Gopi, Christopher R Madan

**Affiliations:** School of Psychology, University of Nottingham, Nottingham, UK

**Keywords:** Metamemory, memory self-efficacy, memory complaints, memory beliefs, memory evaluation

## Abstract

Subjective memory evaluation is important for assessing memory abilities and complaints alongside objective measures. In research and clinical settings, questionnaires are used to examine perceived memory ability, memory complaints, and memory beliefs/knowledge. Although they provide a structured measure of self-reported memory, there is some debate as to whether subjective evaluation accurately reflects memory abilities. Specifically, the disconnect between subjective and objective memory measures remains a long-standing issue within the field. Thus, it is essential to evaluate the benefits and limitations of questionnaires that are currently in use. This review encompasses three categories of metamemory questionnaires: self-efficacy, complaints, and multidimensional questionnaires. Factors influencing self-evaluation of memory including knowledge and beliefs about memory, ability to evaluate memory, recent metamemory experiences, and affect are examined. The relationship between subjective and objective memory measures is explored, and considerations for future development and use of metamemory questionnaires are provided.

Across research and clinical settings, there is a wide range of objective and subjective measures available to examine memory ability (Beaudoin & Desrichard, 2011; Garcia et al., 1998). Objective measures such as recall tests or neuropsychological batteries simulate memory-demanding situations and allow for performance evaluations (Garcia et al., 1998; Schmidt et al., 2001). However, individual differences can influence motivation and performance on memory tasks and objective tests might not accurately reflect naturalistic situations (Bennett-Levy & Powell, 1980; Hultsch et al., 1985; Jopp & Hertzog, 2007; Sander et al., 2018). On the contrary, subjective measures such as questionnaires may better highlight some individual differences specifically regarding self-perceptions about memory and reveal important information about memory in daily life (Herrmann, 1982; Hultsch et al., 1987; Troyer & Rich, 2002).

Structured memory questionnaires were developed to examine the connection between beliefs and behaviour, age-related memory decline, and memory complaints in clinical settings (Cavanaugh et al., 1998; Crook et al., 1992; Herrmann, 1982). In addition, it was expected that questionnaires would provide an efficient alternative to observational field work and a structured ecological approach to assessing everyday memory (Garcia et al., 1998; Herrmann, 1982). Given the complexity of memory, various questionnaires have been devised to examine different types of memory and for specific target groups (Gilewski & Zelinski, 1986; Herrmann, 1982). With numerous questionnaires available, determining the appropriate one to use in a research or clinical setting requires some consideration.

This review provides an overview of common *metamemory* questionnaires that are currently in use across research and clinical settings and highlights factors that should be considered when using subjective measures. The original definition of metamemory encompassed beliefs about one’s own memory ability and factual knowledge about memory processes (Cavanaugh et al., 1998; Dixon & Hultsch, 1983; Flavell & Wellman, 1975). This has been further divided into memory knowledge (factual knowledge about memory processes, tasks, and strategies), monitoring (knowledge about personal memory use), self-efficacy (beliefs about personal memory abilities), and affect (affective states linked to memory situations) (Hertzog et al., 1989; Hultsch et al., 1988).

As metamemory is multidimensional, many questionnaires are encompassed under this term, yet the purpose of assessment and the items used differ and questionnaires are therefore not interchangeable (Cornish, 2000). Here, we focus on three common types of metamemory questionnaires: self-efficacy, complaints, and multidimensional. These are targeted to examining personal beliefs about ability, memory failures or complaints about memory in daily life, or multiple dimensions of metamemory (Beaudoin & Desrichard, 2011; Crumley et al., 2014; Gilewski & Zelinski, 1986). These questionnaires are generally applicable across different populations in research and clinical settings, and thus their characteristics are worth exploring. However, it is important to note these broadly defined categories are not mutually exclusive and have been used interchangeably throughout the literature.

In past research, there has been some ambiguity around the term *memory complaints* and suggestion that perhaps memory complaints can be considered as an aspect of self-efficacy (Hertzog & Pearman, 2013; Jonker et al., 2000). In addition, previous research has demonstrated correlations between complaints and self-efficacy subscales of multidimensional questionnaires (Hertzog et al., 1989; Troyer & Rich, 2002). However, as highlighted by Beaudoin and Desrichard (2011), memory self-efficacy and complaints are distinguished by forgetfulness being related to embarrassment in the context of complaints. Furthermore, as mentioned by Ossher et al. (2013), the lack of correlation they found between the Everyday Memory Questionnaire and Memory Self Efficacy Questionnaire demonstrates that complaints measures recording everyday errors and self-efficacy measures of perceived ability are distinct. Thus, these types of questionnaires have been examined separately here.

Several characteristics were considered for including or excluding questionnaires in this review. We included structured, validated metamemory questionnaires developed for adults that are currently in use and focus on memory self-efficacy, complaints, or knowledge. Questionnaires beyond the scope of this review were those designed for a single study, targeted to one type of memory (e.g., prospective), designed and currently in use with only patient groups (e.g., stroke patients), task-specific predictions/evaluations, checklists, interviews, and open-ended questionnaires. The rationale for these criteria was to clearly outline a list of current, validated, and structured questionnaires within each category that can be used with a wide range of adults in clinical and research settings. Self-efficacy, memory complaints, and multidimensional questionnaires were selected for examination because they have been consistently used throughout past research, yet there is little information available to distinguish the types of questionnaires and their characteristics (Gilewski & Zelinski, 1986; Herrmann, 1982). In addition, some questionnaires were excluded as their focus was too narrow (e.g., for specific patient groups) which may be better examined in comparison to similar patient-focused questionnaires. These criteria allow for an examination of different factors such as subscales, internal consistency, and length that can aid researchers and clinicians in determining which questionnaires to use.

## Self-efficacy questionnaires

Perceptions of one’s own memory ability have been labelled memory self-efficacy (Beaudoin & Desrichard, 2011; Hultsch et al., 1988). This is distinguished from knowledge about memory processes by the personal agency in different circumstances and from memory monitoring with a focus on beliefs about ability rather than reporting memory successes or failures in daily life (Cavanaugh et al., 1998; Hultsch et al., 1988). Self-efficacy questionnaires often include ratings of the ability to remember information in various contexts. Questionnaires currently in use are shown in Table 1.

**Table 1.** Memory self-efficacy questionnaires.

Questionnaire	Revised versions and language validations	General purpose	Subscales (no. of items)	Sample item	Response scale and indications	Internal consistency (a)
SSMQ: Squire Subjective Memory Questionnaire (Squire et al., 1979)	Dutch (Van Bergen et al., 2010); Polish (Kuczek et al., 2018); Chinese (Zhang et al., 2022)	Initially to examine memory complaints before and after electroconvulsive therapy but adapted to general subjective memory evaluation	Ability to remember (18): Perception of ability to use memory in different situations	My ability to remember things that have happened more than a year ago is	Worse than ever before—Better than ever before (9-point) (+ = better ability)	.88–.96
SMQ: Subjective Memory Questionnaire (Bennett-Levy & Powell, 1980)	SMQ-R (Lucas et al., 1991)	Measure subjective memory abilities in adults; Developed with a clinical approach to assessing memory in everyday life	Ability to remember (36): memory ability for specific items/situations	How good is your memory for. . . names of streets/houses	Very bad—Very good (5-point) (+ = better ability)	N/A
			Frequency of forgetting (7): how often items/situations are forgotten	How often do you . . . forget a particular word in mid-sentence	Very rarely—Very often (+ = more lapses)	N/A
MAC-S: Memory Assessment Clinics, Self-Rating Scale (Winterling et al., 1986)	MAC-E (Miller et al., 2022)	Self rating scale for memory based on previous measures	Global (4): general ability and distress related to memory decline	In general, as compared to the average individual, how would you describe your memory?	Very poor—Very good (5-point) (+ = better ability)	N/A
			Ability (21): ability to remember in specific situations	How would you describe your ability to remember. . . where you have put objects in the home or office.	Very poor—Very good (+ = better ability)	N/A
			Frequency (24): difficulty remembering in specific situations	How often would you say you do each of the following? Fail to remember a name or word but recall it later	Very often—Very rarely (+ = fewer lapses)	N/A
MSEQ: Memory Self-Efficacy Questionnaire (Berry et al., 1989)	French (Beaudoin et al., 2008)	Measure of self-efficacy for examining development and individual differences in memory evaluation	Ability to remember across 8 subscales with 5 levels per subscale: Grocery, Phone, Picture, Location, Word, Digit, Map, Errands, Photographs, Maze	If I heard it twice, I could remember 2 items from a friend’s grocery list of 12 items, without taking any list with me to the store	Yes/No followed by confidence judgement (10%–100%) (+ = higher confidence)	.83–.90

Language validations as far as known by authors. Unless otherwise specified, the scale points are consistent throughout the questionnaires and only mentioned in the first subscale.

A prominent self-efficacy questionnaire, the Squire Subjective Memory Questionnaire (SSMQ) was adapted from a memory complaints measure to a more general use questionnaire with a unidimensional structure reflecting beliefs about one’s own memory (Squire et al., 1979; Van Bergen et al., 2010). On the other hand, the Subjective Memory Questionnaire (SMQ), was initially designed to target self-efficacy by assessing self-report of skills in real-life memory tasks for adults. In a different approach, the Memory Assessment Clinics Self-Rating Scale (MAC-S) was developed to address limitations of existing memory scales for use within research and clinical settings (Bennett-Levy & Powell, 1980; Crook & Larrabee, 1990; Winterling et al., 1986). Finally, the commonly used Memory Self-Efficacy Questionnaire (MSEQ) was designed to examine individual differences in memory ability (Berry et al., 1989).

As demonstrated in Table 2, all self-efficacy questionnaires include an ability to remember subscale. These questions examine perceived memory ability for specific information (e.g., names), past actions (e.g., where an item was placed earlier), or hypothetical scenarios (e.g., remembering a shopping list in a grocery store). Although there are differences between the items, response scales, and variations in question framing, self-efficacy questionnaires generally require respondents to evaluate their strengths and weaknesses, in line with the underlying construct of self-efficacy (Hultsch et al., 1988).

**Table 2.** Complaints questionnaires.

Questionnaire	Revised versions and language validations	General purpose	Subscales (no. of items)	Sample item	Response scale and indications	Internal consistency (a)
EMQ: Everyday Memory Questionnaire (Sunderland et al., 1983)	EMQ-28 (Sunderland et al., 1984); EMQ-Revised (Royle & Lincoln, 2008); EMQ-20 (Calabria et al., 2011); EMQ-R (Evans et al., 2020), Swedish (Olsson et al., 2006); Greek (Efklides et al., 2002)	Examine memory failures in everyday life	Failures (35): failures in daily life across speech, reading and writing, faces and places, actions, and learning new things	Forgetting something you were told yesterday or a few days ago.	Never—Several times in a day (5-point) (+ = more failures)	N/A
MAC-Q: Memory Complaint Questionnaire (Crook et al., 1992)	N/A	Brief, reliable measure of memory complaints in clinical or research settings	Memory decline (6): comparative ability to high school	Remembering the name of a person just introduced to you	Much better now—Much poorer now (5-point) (+ = decline)	0.57
SMCS: Subjective Memory Complaints Scale (Schmand et al., 1996)	Portuguese (Mendes et al., 2008)	Examine memory complaints	Complaints (10): memory problems in daily life	Do you have any complaints concerning your memory?	No—Yes, serious problem (4-point) (+ = more complaints)	0.72
FOF-10: Frequency of Forgetting-10 (Zelinski & Gilewski, 2004)	N/A	Short measure for clinical assessments derived from the Frequency of Forgetting MFQ scale	Global (1): general frequency of forgetting	How would you rate your memory in terms of the kinds of problems that you have?	Major problems—No problems (7-point) (+ = fewer problems)	0.80
			Frequency of forgetting (9): frequency of memory problems	How often do these present a problem for you? Where you put something	Always—Never (+ = fewer lapses)	
SMCQ: Subjective Memory Complaints Questionnaire (Youn et al., 2009)	Korean (Youn et al., 2009)	Examine subjective memory complaints in older adults in research and clinical settings	Global memory function (4): general functioning in everyday life	Do you think that your memory is poorer than that of other people of a similar age?	Yes—No (2-point) (+ = higher severity of complaints)	0.69
			Everyday memory functioning (10): difficulty remembering/failures	Do you have difficulty in remembering an appointment made a few days ago?	Yes—No (+ = higher severity of complaints)	0.83
MCS: Memory Complaints Scale (Vale et al., 2012)	Portuguese (Vale et al., 2012)	Actively search for memory complaints	Complaints (7): general problems and frequency	How is your memory compared with when you were younger?	Same or better—Much worse (3-point) (+ = more complaints)	0.85

MFQ: Memory Functioning Questionnaire.

Language validations as far as known by authors. Unless otherwise specified, the scale points are consistent throughout the questionnaires and only mentioned in the first subscale.

There are also differences across the scales. The SSMQ includes questions about attention and perception, which can result in respondents confounding memory with other cognitive domains (Squire et al., 1979). However, it is relatively short, which can be advantageous for populations where fatigue is a concern. While the SMQ and MAC-S include frequency of forgetting subscales, only the MAC-S includes a global subscale which could be useful in understanding an individual’s perception of their general memory functioning (Bennett-Levy & Powell, 1980; Crook & Larrabee, 1990). The MAC-S also has subsections of the ability subscale for different types of memory such as everyday task-oriented memory and remote memory (Winterling et al., 1986). In a different capacity, the MSEQ incorporates self-efficacy strength and level with confidence ratings and varying task difficulty levels, which is beneficial for examining nuances in self-efficacy ratings (Berry et al., 1989).

The outlined self-efficacy questionnaires have been used across different populations to examine memory distrust, help-seeking behaviours, memory training, and the link between memory self-efficacy and memory performance (Beaudoin & Desrichard, 2017; D’Angelo et al., 2021; Gigi et al., 2020; Van Bergen et al., 2010). These questionnaires can also be helpful in examining the relation between perceived self-efficacy and motivation for participating in memory-demanding tasks (Hertzog et al., 1990; Ponds & Jolles, 1996). Although they share some characteristics, these scales are not interchangeable, and their differing characteristics are worth evaluating in relation to the purpose of assessment in research and clinical settings. Finally, it has been suggested that self-efficacy measures likely reflect beliefs rather than actual abilities and this should be considered when selecting a questionnaire for use (Herrmann, 1982; Ossher et al., 2013).

## Complaint questionnaires

Memory complaints have been broadly defined as reports of problems or failures in daily life, usually from older adults (Gilewski & Zelinski, 1986; Hertzog & Pearman, 2013). Complaints should be seriously considered by clinicians as they may be predictive of cognitive decline or dementia (Reid & MacLullich, 2006; Schmand et al., 1996). Complaints questionnaires systematically assess memory failures or problems in daily life, often focusing on the frequency of forgetting. Questionnaires currently in use are shown in Table 2.

A well-established questionnaire, the Everyday Memory Questionnaire (EMQ), was initially designed to examine memory failures for head-injured patients but has been widely adopted across different populations (Calabria et al., 2011; Sander et al., 2018; Sunderland et al., 1983). In a different capacity, the Memory Complaint Questionnaire (MAC-Q) and the Subjective Memory Complaints Scale were developed to examine memory decline and complaints particularly for older adults (Crook et al., 1992; Schmand et al., 1996). These questionnaires have been used as brief, reliable measures to assess severity of complaints and the relationship between complaints and objective performance (Buckley et al., 2013; Mendes et al., 2008).

The Frequency of Forgetting-10 (FoF-10) was derived from the Frequency of Forgetting Scale of a multidimensional questionnaire as a brief measure to be used in clinical settings (Zelinski & Gilewski, 2004). Although the authors describe this questionnaire as a self-efficacy measure, here it is considered a complaints measure as it records memory problems in everyday life rather than perceived ability (Zelinski & Gilewski, 2004). Two more recently developed questionnaires, the Subjective Memory Complaints Questionnaire (SMCQ) and the Memory Complaints Scale (MCS) were specifically targeted at assessing memory complaints (Vale et al., 2012; Youn et al., 2009). These questionnaires have been used to examine memory complaints in patient groups, the relationship between complaints and objective measures, and for screening for mild cognitive impairment (Dalpubel et al., 2019; Huang et al., 2014; Yim et al., 2017).

As shown in Table 2, memory complaint scales focus on the frequency of forgetting, memory decline, and complaints about memory functioning. The FoF-10 and SMCQ also contain global subscales to evaluate whether respondents believe that there are general memory problems (Youn et al., 2009; Zelinski & Gilewski, 2004). Memory complaint questionnaires reflect an individual’s calculation of how often particular mistakes are made in daily life, which relies on memory monitoring, one of the dimensions of metamemory outlined above (Hultsch et al., 1988). However, as highlighted by Hertzog et al. (1989), it is possible that the frequency of forgetting is more likely to be a combination of recent experiences and beliefs about memory than an accurate search of memory experiences.

There are several differences across complaints questionnaires worth noting. The EMQ broadly differentiates between memory and attention systems, which is beneficial to separately address domain-specific difficulties (Royle & Lincoln, 2008; Sunderland et al., 1983). In contrast to the other questionnaires, the MAC-Q examines memory change by comparing current memory ability to that of high school or college (Crook et al., 1992). However, this measure has been found to be impacted by affective status and caution has been advised when using this questionnaire with older adults (Reid et al., 2012).

While the EMQ can be administered to both patients and relatives, the MCS is the only measure that contains a separate companion version (MCS-B) and also examines the impact of memory problems on daily activities (Sunderland et al., 1983; Vale et al., 2012). This can be beneficial where memory impairment impacts the ability to respond and understand the real-world implications of memory problems (Garcia et al., 1998; Sugden et al., 2022; van der Werf & Vos, 2011). Finally, there are different response scales, target items, and questionnaire lengths across these measures (Table 2). These differences highlight that complaints questionnaires are not interchangeable and considerations regarding the target group and assessment purpose are essential when selecting a questionnaire for use. Although they are beneficial, complaints questionnaires should be interpreted cautiously as measures of noticeable memory failures rather than a veridical account of memory failures (Ossher et al., 2013).

## Multidimensional questionnaires

As mentioned above, metamemory is a multidimensional construct. The self-efficacy and monitoring constructs are examined using self-efficacy and complaints questionnaires yet there are few questionnaires that directly explore memory knowledge or affect in the same targeted approach (Cherry et al., 2000). However, these factors are sometimes included in other questionnaires as subscales. In particular, multidimensional questionnaires examine multiple aspects of metamemory in a single measure (Dixon & Hultsch, 1983). Questionnaires currently in use are shown in Table 3.

**Table 3.** Multidimensional questionnaires.

Questionnaire	Revised versions & language validations	General purpose	Subscales (no. of items)	Sample item	Response scale and indications	Internal consistency (a)
SMS: Sehulster Memory Scale (Sehulster, 1981)	N/A	Examine perceptions of memory across 20 memory-related domains and nine memory experiences	Ability (20): perception of everyday ability	I remember conversations that I had with other people	Very poorly—Extremely well (5-point) (+ = better ability)	N/A
			Frequency of forgetting (20): frequency of items/information forgotten	I forget anniversaries, appointments, birthdays, etc.	Very often—Rarely (+ = fewer lapses)	N/A
			Social comparison (20): memory ability compared with others	I trust my memory more than I trust the memory of	Very few people—All or most people (+ = better personal ability)	N/A
MIA: Metamemory in Adulthood (Dixon et al., 1988)	MIA-Revised (McDonough et al., 2019); Arabic (Alquraan & Aljarah, 2011), Dutch (Ponds & Jolles, 1996), French (Baillargeon & Neault, 1989), Portuguese (Campelo et al., 2016), Japanese (Ide et al., 1999)	Examine metamemory in adulthood	Strategy (18): reported use of internal and external strategies	Do you write yourself reminder notes?	Never—Always (5-point) (+ = high use)	.82–.86
			Task (16): knowledge of memory processes	Familiar things are easier to remember than unfamiliar things	Agree strongly—Disagree strongly (+ = high knowledge)	.78–.83
			Capacity (17): beliefs about own memory capacity	I am good at remembering names	Agree strongly—Disagree strongly (+ = high capacity)	.81–.86
			Change (18): perceived change in remembering capacity	The older I get the harder it is to remember things clearly	Agree strongly—Disagree strongly (+ = stability)	.90–.93
			Anxiety (14): perception of the relationship between anxiety and memory performance	I find it harder to remember things when I’m upset	Agree strongly—Disagree strongly (+ = high knowledge)	.83–.87
			Achievement (16): perception of own motivation to perform well on memory tasks	It is important to me to have a good memory	Agree strongly—Disagree strongly (+ = high achievement)	.76–.79
			Locus (9): perceived control over memory ability	I have little control over my memory ability	Agree strongly—Disagree strongly (+ = internality)	.71–.78
MFQ: Memory Functioning Questionnaire (Gilewski et al., 1990)	FOF-10 (Zelinski & Gilewski, 2004); Italian (Pedone et al., 2005); Turkish (Şahin et al., 2013)	Developed by the same researchers of the Metamemory Questionnaire to examine subjective memory compared to objective performance	General rating (1): global memory problems	How would you rate your memory in terms of the kinds of problems that you have?	Major problems—No problems (7-point) (+ = higher perceived functioning)	General Frequency of forgetting factor: .94
			Frequency of forgetting (18): frequency of forgetting incidents	How often do these present a problem for you? personal dates (e.g., birthdays)	Always—Never (+ = fewer forgetting incidents)	
			Frequency of forgetting when reading (10): frequency of forgetting incidents while reading	As you are reading a novel, how often do you have trouble remembering what you have read . . . the chapter before the one you are currently reading	Always—Never (+ = fewer forgetting incidents)	
			Remembering past events (4): perceived functioning for past information/events	How well you remember things that occurred . . . last month is	Very bad—Very good (+ = higher perceived functioning)	
			Seriousness of forgetting (18): seriousness of forgetting incidents	When you actually forget in these situations, how serious of a problem do you consider the failure to be? . . . Appointments	Very serious—Not serious (+ = less serious incidents)	0.94
			Retrospective functioning (5): comparative functioning to past self	How is your memory compared to the way it was . . . when you were 18?	Much worse—Much better (+ = improvement in functioning)	0.89
			Mnemonics usage (8): use of internal and external aids	How often do you use these techniques to remind yourself about things? . . . mental repetition	Always—Never (+ = less use of mnemonics)	0.83
PBMI: Personal Beliefs about Memory Instrument (Lineweaver & Hertzog, 1998)	PBMI Computerised (Hertzog et al., 2014); Japanese (Kinjo & Shimizu, 2014)	Measure beliefs about one’s own memory	Global memory self ability (1): global ability	My ability to remember in general is	Very poor—Very good (Continuous) (+ = better ability)	N/A
			Relative standing ratings (2): comparitive scale to adults of same age	Compared to all adults my age, my memory is:	Much below average—Much above average (+ = better comparative ability)	N/A
			Retrospective change (7): comparative functioning to past self	Over the last 10 years, my memory has	Greatly declined—Greatly improved (+ = improvement)	0.96
			Prospective change (7): expected memory change in the future	When I am 75 years old, my memory will be	Much less efficient—Much more efficient (+ = predicted improvement)	0.97
			Control (4): perceived control over abilities	There are things I can do to help me remember	Strongly disagree—Strongly agree (+ = more control)	0.93
			Prospective control (9): perceived control over change	I can control the amount my memory will change in the future.	Strongly disagree—Strongly agree (+ = more control)	0.90
			Future control (5): perceived control over future abilities	In the future, the amount of control I will have over my memory will be	None—A lot (+ = more control)	0.83
			Specific memory ability (23): perceived ability in different situations	Daily Schedule: My ability to remember my daily schedule is	Very poor—Very good (+ = better ability)	0.94
GBMI: General Beliefs about Memory Instrument (Lineweaver & Hertzog, 1998)	GBMI Computerised (Hertzog et al., 2014); Japanese (Kinjo & Shimizu, 2014)	Measure beliefs about memory for an average healthy adult	Global memory ability (1): global ability of healthy adults	Ability to remember in general.	Very poor—Very good (Continuous) (+ = better ability)	N/A
			Control (1): amount of control adults have over their memory ability	Amount of control over present memory functioning. The extent to which the adult can do things now that will determine how his or her memory works.	No control—Complete control (+ = better control)	N/A
			Prospective Control (1): amount of control adults have over future ability	Amount of control over future memory functioning. The extent to which the adult can do things now that will determine how his or her memory will work in the future.	No control—Complete control (+ = better control)	N/A
			Specific memory ability (12): memory ability for adults in different situations	Ability to remember trivia	Very poor—Very good (+ = better ability)	N/A
MMQ: Multifactorial Memory Questionnaire (Troyer & Rich, 2002)	Languages: Dutch (van der Werf & Vos, 2011); French (Fort et al., 2004), Italian (Raimo et al., 2016)	Assess self-reported memory for use in clinical assessments	Contentment (18): satisfaction with memory ability	I am embarrassed about my memory ability.	Strongly agree—Strongly disagree (5-point) (+ = greater contentment)	0.95
			Ability (20): perception of everyday ability	How often do you leave something behind when you meant to bring it with you?	All the time—Never (+ = better subjective ability)	0.93
			Strategy (19): everyday use of strategies and memory aids	How often do you create a rhyme out of what you want to remember?	Never—All the time (+ = more frequent use of strategies)	0.83

Language validations as far as known by authors. Unless otherwise specified, the scale points are consistent throughout the questionnaires and only mentioned in the first subscale.

One of the early multidimensional questionnaires, the Sehulster Memory Scale, was designed to examine multiple domains of memory and experiences within a self-theory of memory framework (Sehulster, 1981). Similarly, two of the most prominent multidimensional questionnaires are the Metamemory in Adulthood Questionnaire (MIA), designed to capture aspects of memory relevant to everyday activities and the Memory Functioning Questionnaire (MFQ), developed to examine memory abilities and failures (Dixon et al., 1988; Gilewski et al., 1990). In contrast, Lineweaver and Hertzog (1998) developed two measures to separate beliefs about one’s own memory, with the Personal Beliefs about Memory Inventory (PBMI), from beliefs about the memory ability of people in the general population, using the General Beliefs about Memory Inventory (GBMI). The more recently developed Multifactorial Memory Questionnaire (MMQ) was designed to tap three aspects of memory: contentment, ability, and strategy use (Troyer & Rich, 2002).

As shown in Table 3, these questionnaires generally have multiple subscales, with some that are shared across measures. For example, global ability, self-efficacy, control, and mnemonics/strategy scales are included in several questionnaires. These shared characteristics highlight the multidimensional nature of memory and the importance of multiple scales to effectively assess knowledge and perceptions of individual ability (Dixon & Hultsch, 1983; Mogle et al., 2021). However, similar subscales are not interchangeable. For example, although the MIA and MMQ include affect-related subscales, the MIA anxiety subscale examines the ability to use memory when in different emotional states, whereas the MMQ contentment subscale is more targeted to satisfaction with memory ability (Hultsch et al., 1988; Troyer & Rich, 2002). Finally, although there has been some consideration regarding separating internal (e.g., mnemonics) and external (e.g., writing down reminders) memory aids in strategy subscales, these are often included together within one subscale (e.g., MIA Strategy, MFQ Mnemonics, MMQ Strategy; see Table 3) (Dixon & Hultsch, 1983; Dixon et al., 1988; Gilewski et al., 1990; Troyer & Rich, 2002).

The differences across questionnaires should also be noted. In contrast to the other questionnaires, the MIA examines knowledge about memory processes and the perceived importance of good memory, whereas the MFQ examines the perceived seriousness of forgetting (Dixon et al., 1988; Gilewski et al., 1990). On the other hand, the PBMI includes a relative standing rating where respondents are asked to compare their general memory ability to all adults of their own age and all adults of all ages (Lineweaver & Hertzog, 1998). These and other unique characteristics such as different rating scales and questionnaire lengths are important to consider when selecting a questionnaire to use. For example, if the frequency of forgetting when reading is important, the MFQ would be appropriate, whereas others without this subscale may not be as valuable. These distinct subscales also support the argument that there is still work to be done in dissecting the components of the multidimensional construct of metamemory (Hultsch et al., 1987; Mogle et al., 2021).

These multidimensional questionnaires have been used to examine the interrelationships between metamemory dimensions, aging, memory strategies in daily life, and the relationship between subjective evaluations and objective memory performance (Cavallini et al., 2013; Hertzog et al., 1989; Irak & Çapan, 2018; Macan et al., 2010; Payne et al., 2017). However, they have also been criticised for their lengthy time to complete, which may deter use with certain populations due to fatigue and consequently incomplete or inaccurate responses (Troyer & Rich, 2002; Van Bergen et al., 2010). Thus, some questionnaires have been reduced to shorter versions or specific subscales to retain essential aspects while reducing the length (Campelo et al., 2016; McDonough et al., 2019; Zelinski & Gilewski, 2004). In contrast, the MMQ was designed with considerations for patient groups and has been successfully applied to clinical populations (Illman et al., 2015; van der Werf & Vos, 2011).

Another concern is that respondents may rely on generalisations or stereotypes when responding to global questions, whereas they may consider distinct past experiences when responding to anchored questions (Cyr & Anderson, 2019; Mogle et al., 2020). This can potentially result in inaccurate evaluations depending on the order of questions (Cyr & Anderson, 2019). On the other hand, it has been suggested that multidimensional questionnaires might be more closely related to objective memory performance and provide much more detail about an individual’s memory in daily life compared to single questions, interviews, or unidimensional questionnaires (Crumley et al., 2014). Thus, consideration of the benefits and limitations of using longer questionnaires is warranted especially for older adults and patient groups (Davis et al., 1995).

## Questionnaires beyond the scope of this review

Several metamemory questionnaires are not within the scope of this review. For example, the Short Inventory of Memory Experiences has been mentioned in past reviews, but appears to be rarely used and with limited psychometric properties or scale items available (Gilewski & Zelinski, 1986; Herrmann, 1982; Herrmann & Neisser, 1978). Scales such as the Memory Controllability Inventory and the Cognitive Failures Questionnaire are commonly used but are not within the scope of the categories outlined above (Broadbent et al., 1982; Lachman et al., 1995). However, some patient groups and specific memory domain questionnaires might be of interest to researchers and clinicians examining self-reported memory, and we have provided a brief outline of some questionnaires in the online Supplementary material.

## Factors influencing self-evaluation of memory

There are individual differences in the way memory is evaluated and therefore reported on questionnaires (Cavanaugh et al., 1998; Sehulster, 1981; Van Bergen et al., 2009). Several factors may interact with memory self-evaluations including knowledge or beliefs about memory, ability to evaluate memory, recent metamemory experiences, and affect (Hertzog et al., 1989; Rowell et al., 2016; Troyer, 2001). These factors are considered below.

### Knowledge and beliefs about memory

An individual’s conceptualisation of memory and beliefs about stereotypes and control over memory can influence self-evaluations (Cavanaugh et al., 1998; Troyer & Rich, 2002). For example, memory self-reports have been correlated with different cognitive functions such as attention, language, and executive function (Hülür et al., 2018; Jopp & Hertzog, 2007; Snitz et al., 2015). This is perhaps due to the inclusion of attention and executive functioning items in questionnaires (Crook et al., 1992). To prevent respondents from considering these cognitive functions under the umbrella term of memory, a definition of memory that corresponds with the questionnaire can be included in the instructions (Mogle et al., 2020).

Although some individuals are knowledgeable about memory processes, they can still rate their own memory abilities as poor based on generalisations or stereotypes (Cavanaugh et al., 1998; Troyer, 2001). A prominent stereotype is that memory will inevitably decline with age (Cavallini et al., 2013; Hess et al., 2003; Kahn et al., 1975). Consequently, older adults may underestimate their memory ability, whereas younger adults overestimate their memory ability (Cavanaugh et al., 1998; Fritsch et al., 2014). In addition, regular lapses in daily life might be attributed to cognitive decline for older adults, whereas younger adults find them to be insignificant (Zarit et al., 1981). Furthermore, stereotypes may influence performance on objective measures whereby older adults expect to perform poorly, resulting in a lack of motivation and subsequently poor performance (Bouazzaoui et al., 2020; Poon et al., 1978; Troyer & Rich, 2002).

Like beliefs about aging, beliefs about the malleability of memory may influence self-evaluation and objective memory performance (Lachman et al., 1995; Thana-Udom et al., 2021). Perceptions that memory decline is unchangeable can result in negative self-evaluations and reduce motivation to participate in memory-demanding tasks (Bouazzaoui et al., 2020; Cavanaugh et al., 1998). Specifically, older adults may believe memory decline to be inevitable and irreversible, whereas young adults may view their memory to be more malleable, with more control over their abilities (Cavallini et al., 2013; Dixon & Hultsch, 1983; Lineweaver & Hertzog, 1998). On the other hand, the belief that memory is malleable may result in motivation to improve memory abilities and more positive reflections of ability (Bouazzaoui et al., 2020; Cavanaugh et al., 1998). Perhaps education about memory processes, strategies, and normative levels of performance on particular tasks can improve memory control beliefs and mitigate the effects of stereotypes (Hertzog et al., 1990; Lineweaver et al., 2023; Thana-Udom et al., 2021).

### Ability to evaluate memory

Responding to questionnaires requires self-awareness of abilities and recent memory experiences (Davis et al., 1995; Snitz et al., 2015). Memory deficits can affect this retrieval and result in over or under-estimation of abilities (Clare et al., 2010; Helmstaedter & Elger, 2000; Hultsch et al., 1985). Young and middle-aged adults are potentially more accurate in assessing their memory abilities than older adults because they can update their self-evaluations based on regular feedback through work or education (Van Bergen et al., 2009, 2010). On the other hand, older adults or memory-impaired individuals might be more attuned to memory failures in everyday life due to general expectations of memory decline or impairment and may therefore be more realistic in estimations (Crumley et al., 2014; Van Bergen et al., 2010).

Potential solutions to the difficulty of evaluating one’s own memory include questionnaires for companions or checklists and diaries that capture daily remembering instances (Garcia et al., 1998; Sugden et al., 2022). However, memory may be assessed differently depending on whether it is being assessed for oneself or another person, and accurate reporting would require regular contact with the individual to observe memory successes and failures (Helmstaedter & Elger, 2000; Sugden et al., 2022). Furthermore, discrepancies between self and other reports and objective memory measures indicate a lack of reliability in determining which approach best represents actual memory abilities (Helmstaedter & Elger, 2000; Sugden et al., 2022).

Individuals may also differentially evaluate the severity of memory failures. For example, forgetting keys may be considered a failing memory for older adults but a rectifiable lapse for younger adults. As highlighted by Burmester et al. (2015), open-ended questions prior to a structured questionnaire might elucidate the most distressing memory complaints. However, this might not always be feasible within clinical settings due to time limitations and having a global memory question might result in memory worries and salience of negative experiences when responding to more specific questions (Cyr & Anderson, 2019). For example, should a patient report their most distressing memory problems first, they may then exaggerate the severity of other problems on a later questionnaire.

### Recent metamemory experiences

Past experiences, information provided by others, or comparisons of personal performance can influence memory evaluations (Flavell & Wellman, 1975; Hertzog et al., 1989; Sehulster, 1981). For example, if responding to a question about remembering names, an individual may recall a recent event in daily life involving recalling names (Pearman & Trujillo, 2013; Sehulster, 1981). Similarly, memory complaints may partially reflect a comparison with one’s own performance in the past or to others of a similar age (Sehulster, 1981; Zarit et al., 1981). In a different capacity, when reflecting on recent experiences some individuals may consider the use of external or internal memory aids as an extension of their own memory, whereas others evaluate their memory based on performance without aids (Sander et al., 2018).

In a broader scope, frequent forgetting incidents that cause embarrassment or frustration may lead to negative perceptions of general memory ability (Hertzog et al., 1989; Poon et al., 1978). In addition, older adults who have been recently exposed to individuals with dementia could consequently consider their own normal age-related memory decline as indicators of dementia (Kinzer & Suhr, 2016; Van Bergen et al., 2009). On the other hand, this could also result in updated beliefs about what constitutes good memory and may lead to more positive self-evaluations (Pearman et al., 2014). Similarly, older adults may consider recent memory lapses to be part of normal aging (Mogle et al., 2019; Pearman et al., 2014).

Recollection of past experiences can also be influenced by question format. Some items require consideration of recent events, others ask about prior years or decades, and some do not include an anchor (Mogle et al., 2020; Rowell et al., 2016). This can lead to different levels of specificity during retrieval and reliance on generalisations or stereotypes about more remote periods (Cyr & Anderson, 2019; Hultsch et al., 1987). However, older adults might be more accurate when recalling earlier life events, whereas younger adults may be more accurate when retrieving recent events (Kahn & Miller, 1978; Kahn et al., 1975). As such, Kahn and Miller (1978) suggested that a more appropriate comparison would be to ask about a similar event recently and several years prior.

### Affect

Temporary or chronic affective states including stress, anxiety, and depression can influence memory evaluations (Cavanaugh et al., 1998; Rowell et al., 2016; West et al., 1984). Negative feelings towards oneself before performing a memory task or responding to questionnaires may result in poorer outcomes on both (Bouazzaoui et al., 2020; Poon et al., 1978; Riege, 1983). Affect may also impact motivation to participate in memory-demanding tasks and seek help for memory problems (Gigi et al., 2020; Pires et al., 2012). On the other hand, memory problems, failures, or concerns about decline can influence anxiety and depression (Bhang et al., 2020; Mogle et al., 2019; West et al., 1984).

Past research has consistently demonstrated a link between affect and subjective memory evaluations (Buckley et al., 2013; Lineweaver & Brolsma, 2014; Rowell et al., 2016; West et al., 1984). Scores on memory questionnaires are significantly correlated with scores on anxiety and depression measures across healthy adults and patient groups (Hall et al., 2009; Rowell et al., 2016; Yoon et al., 2019). However, evidence is equivocal regarding the link between affect and memory performance (Bouazzaoui et al., 2020; West et al., 1984). It has been suggested that memory evaluations may relate to affective states rather than actual ability, and therefore screening for affective status is worthwhile when examining memory (West et al., 1984; Yoon et al., 2019). In addition, personality traits such as neuroticism and conscientousness have been linked to memory evaluations (Pearman et al., 2014; Zelinski & Gilewski, 2004). Thus, perhaps both affect and personality traits are worth considering alongside memory complaints.

## Relationship between subjective and objective memory measures

There is little consistency across previous research on the relationship between subjective memory evaluation and objective performance (Crumley et al., 2014; Reid & MacLullich, 2006). Some studies have found no correlation, whereas others have found only weak or moderate correlations between measures (Burmester et al., 2017; Schmidt et al., 2001). Meta-analyses have consistently revealed weak correlations between subjective and objective memory measures (*r* < .20) (Beaudoin & Desrichard, 2011; Crumley et al., 2014). There are several possible reasons for this discrepancy.

The low ecological validity of objective measures may influence the relationship between subjective and objective measures. Objective memory tests often have an element of learning information to be later tested which does not reflect learning in real life and is usually conducted in an environment with minimal to no distractions (Herrmann, 1982; Sander et al., 2018). In contrast, questionnaires prompt retrieval of past experiences and general ability (Ossher et al., 2013; Troyer & Rich, 2002). Even “ecologically valid” objective tests such as remembering a shopping list will differ in test settings compared to real life (Crumley et al., 2014). This is evidenced by nonsignificant or weak relationships between tests such as the Weschler Memory Scale and the Rivermead Behavioural Memory Test and subjective measures (Hall et al., 2009; Schmidt et al., 2001).

While external memory aids can be used in everyday situations, memory testing in lab situations often precludes this option (Bennett-Levy & Powell, 1980; Ossher et al., 2013; Troyer & Rich, 2002). Yet including the use of external aids in lab-based testing would introduce measurement issues regarding ceiling effects and not being able to truly measure objective memory (Crumley et al., 2014). Furthermore, when comparing subjective and objective memory, incompatible tests are often used where the objective measures have a little reflection of memory tasks in everyday life (Davis et al., 1995; McMillan, 1984; Ponds & Jolles, 1996).

Another possible reason for the discrepancy is that subjective and objective measures may be examining different aspects of memory (Hall et al., 2009; Herrmann, 1982; Hultsch et al., 1985). While objective tasks require conscious recollection of information, questionnaires can invite retrieval of semantic information about memory ability and stereotypes (Schmidt et al., 2001). For example, recall and recognition tests require concentrated learning of specific information with anticipated testing, whereas questionnaire items ask about the ability to remember or frequency of forgetting in various everyday situations (McMillan, 1984; Ossher et al., 2013; West et al., 1984). Thus, objective measures measure memory ability in a learning and lab-based context, whereas subjective measures examine ability in everyday life (Crumley et al., 2014; Schmidt et al., 2001; West et al., 1984).

Finally, inaccurate reporting may influence the discrepancy between subjective and objective measures (Hall et al., 2009). Questionnaires generally rely on respondents’ interpretation of concept meanings and question understanding, where varying perceptions might influence outcomes (Mogle et al., 2021; Sunderland et al., 1983; Vogel et al., 2016). In addition, individuals relying on stereotypes might negatively evaluate their memory ability even if objective performance is within a normal range (Kahn et al., 1975; Pearman et al., 2014; Troyer & Rich, 2002). Similarly, individuals with cognitive difficulties may underestimate or overestimate memory abilities if they are unable to accurately assess their abilities (Hall et al., 2009; Illman et al., 2015). In a different capacity, confounding memory with other cognitive domains can result in over-reporting of problems such as memory difficulties (Burmester et al., 2017; Hall et al., 2009).

The discrepancy between subjective and objective measures has important implications for the diagnosis and treatment of memory problems and the use of questionnaires. It has been argued that subjective reports cannot be used as a substitute for objective measures and that caution is warranted for use in clinical settings (Crumley et al., 2014; Garcia et al., 1998; Herrmann, 1982). However, it can also be argued that objective reports alone are not sufficient to examine everyday memory, whereas self-report measures uncover important individual differences and information regarding an individual’s concerns and feelings about their memory (Sugden et al., 2022; Troyer & Rich, 2002; van der Werf & Vos, 2011). Perhaps the best approach would then be to combine these measures in creating a memory profile (Herrmann, 1982; Poon et al., 1978).

## Conclusion and future considerations

This review focused on three types of metamemory questionnaires. Self-efficacy questionnaires examine perceived ability in specific situations, complaints questionnaires assess memory difficulties in everyday life, and multidimensional questionnaires address multiple distinct aspects of metamemory. Individual differences such as knowledge/beliefs about memory, ability to accurately evaluate memory, recent metamemory experiences, and affect can influence memory self-evaluations. Moreover, there are often discrepancies between reports and objective memory performance. Thus, several considerations arise for the design and use of memory questionnaires.

Many questionnaires were developed before the commonplace use of assistive technologies (e.g., mobile phones) and therefore warrant caution where questions about external memory aids exclude technology (Miller et al., 2022; Mogle et al., 2020). In addition, some questionnaires have been criticised for limited relevance to clinical use where they include questions regarding public speaking, driving, and working that may be irrelevant to patients (Sander et al., 2018; Troyer & Rich, 2002). For example, questions derived from professional colleagues of researchers or health care professionals might not be applicable to patient groups who face different daily memory difficulties (Bennett-Levy & Powell, 1980; Youn et al., 2009). Perhaps *not applicable* options might be helpful in determining where patient groups and healthy adults diverge in everyday memory problems (Sander et al., 2018). Thus, when developing memory questionnaires, it is essential to carefully consider the target respondents and the appropriate questions that would be relevant to their daily lives (Burmester et al., 2015; Vogel et al., 2016).

When selecting a questionnaire, it is important to consider the target respondents’ memory abilities. Although there is overlap among questionnaires, they are not interchangeable and invite different considerations when responding (Vogel et al., 2016). For example, multidimensional questionnaires are lengthy and may not be beneficial where a particular domain or aspect of memory is the focus (Crook et al., 1992; Troyer & Rich, 2002). Thus, when fatigue or memory impairment may impact responding, perhaps shortened versions or companion reports can be used (Garcia et al., 1998; Vogel et al., 2016). On the other hand, brief questionnaires might not sufficiently reveal the depth of memory deficits in the same way as multidimensional questionnaires (McDonough et al., 2019; Mogle et al., 2021; Reid & MacLullich, 2006). Thus, there is a consideration to be made regarding length, accuracy, and the target respondents’ abilities.

When evaluating questionnaire responses, it is worth considering how individual differences influence memory evaluations and thus rehabilitation or training. While some individuals respond based on past experiences to questionnaires using implicit theories about memory, others respond based on stereotypes or generalisations (Lineweaver & Hertzog, 1998; Pearman et al., 2014). In addition, as outlined earlier, various individual difference factors can influence self-evaluations and vice versa. For example, while self-efficacy measures might be influenced by general perceptions, schemas, and stereotypes, complaint measures might be influenced by situation-specific outcomes (Ossher et al., 2013). Therefore, it is essential to consider responses on questionnaires with some level of caution.

In summary, metamemory questionnaires provide a standardised approach to examine memory beliefs, abilities, and complaints. At present, the consensus is that subjective and objective measures should be combined to provide the most accurate memory profile. Memory questionnaires, if selected appropriately for the target audience and assessment purpose, are essential tools in research and clinical settings.

## Supplemental Material

sj-docx-1-qjp-10.1177_17470218231183855 – Supplemental material for Subjective memory measures: Metamemory questionnaires currently in use Supplemental material, sj-docx-1-qjp-10.1177_17470218231183855 for Subjective memory measures: Metamemory questionnaires currently in use by Yashoda Gopi and Christopher R Madan in Quarterly Journal of Experimental Psychology
