# Supplementary material for: Subjective memory measures: Metamemory questionnaires currently in use
Source: Q J Exp Psychol (Hove). 2023 Jul 3;77(5):924–42. doi: 10.1177/17470218231183855 (PMC11032637; doi:10.1177/17470218231183855)
Supplement: sj-docx-1-qjp-10.1177_17470218231183855 – Supplemental material for Subjective memory measures: Metamemory questionnaires currently in use [file sj-docx-1-qjp-10.1177_17470218231183855.docx]

Supplementary Material for:

**Subjective memory measures: metamemory questionnaires currently in use**

Yashoda Gopi^†^ & Christopher R. Madan

School of Psychology, University of Nottingham, Nottingham, UK

^†^ Corresponding author.

Yashoda Gopi

School of Psychology, University Park

University of Nottingham

Nottingham, NG7 2RD, UK

Email: [yashoda.gopi@nottingham.ac.uk](mailto:yashoda.gopi@nottingham.ac.uk)

***Questionnaires Beyond the Scope of This Review***

Different questionnaires from the ones outlined above have been developed to address various aspects of memory or cognition in healthy adults and clinical populations. For example, questionnaires have been designed for use in educational settings or specific studies and examining cognitive failures or the impact of memory changes on daily life (Broadbent et al., 1982; Gilewski & Zelinski, 1986; Herrmann, 1982; Kahn et al., 1975; Shaikh et al., 2019; Zarit et al., 1981). In addition, there are specific patient groups and autobiographical memory questionnaires (Davis et al., 1995; Palombo et al., 2013; Smith et al., 2000; Sutin & Robins, 2007). Although an extensive evaluation is not within the scope of this review, a brief outline of some patient group and autobiographical memory questionnaires is provided here.

*Patient Groups Questionnaires*

Several questionnaires have been developed to examine subjective memory for dementia and acquired brain injury patients. To screen for Alzheimer’s disease, Koss et al. (1993) developed the Short-Memory Questionnaire (Short MQ), which was derived from the Memory Self-Report Questionnaire (Riege, 1983). The Short MQ has been incorporated in clinical settings to distinguish between individuals with Alzheimer’s disease and healthy older adults (Garcia et al., 1998; Maki et al., 2000; Montgomery et al., 2018).

To examine the frequency of retrospective and prospective memory errors of Alzheimer’s patients, their carers, and healthy adults, Smith et al. (2000) developed the Prospective and Retrospective Memory Questionnaire (PRMQ). The PRMQ is used to examine relationship between subjective evaluation and objective performance and prospective and retrospective memory ability across patient populations (Hogan et al., 2021; Kliegel & Jäger, 2006; Vaskivuo et al., 2018). To examine memory problems in everyday life that may impact older stroke patients, the Subjective Memory Assessment Questionnaire was developed, but is now considered to have poor reliability (Davis et al., 1995; Salis et al., 2019).

A different questionnaire, the Brief Assessment of Prospective Memory, a shortened version of the Comprehensive Assessment of Prospective Memory, was created for use with traumatic brain injury patients but has been expanded for use with stroke patients as well (Hogan et al., 2021; Man et al., 2011, 2015). In a broader scope, the Evaluation of Everyday Memory Questionnaire was created to capture everyday memory instances of acquired brain injury patients and includes a companion version (Tropp et al., 2015). These questionnaires target specific groups, but some have been extended for use with other patient groups. In addition, these questionnaires tend to be relatively short as they target groups who may not be able to engage with longer measures (Sugden et al., 2021). Evidently, memory problems vary across populations, which should be considered when designing and selecting questionnaires for use.

*Autobiographical Memory Questionnaires*

Autobiographical questionnaires examine memory for past personal events (Clark & Maguire, 2020). A well-established questionnaire, the Memory Characteristics Questionnaire (MCQ), was designed to examine ratings of memory characteristics such as visual detail, spatial and temporal information, and feelings (Johnson et al., 1988). Similar to the MCQ, the Autobiographical Memory Questionnaire (AMQ) was derived from autobiographical and memory theories to examine the phenomenological characteristics of autobiographical memories including recollection, vividness, and belief in the memory’s accuracy (Rubin et al., 2003; Talarico et al., 2004).

Another widely used questionnaire, the Memory Experiences Questionnaire (MEQ), was developed to examine the dimensions of autobiographical memory with a shortened version for time-limited situations (Luchetti & Sutin, 2016; Sutin & Robins, 2007). To examine individual differences in trait mnemonics, the Survey of Autobiographical Memory (SAM) was created with several subscales and a brief version developed for epidemiological research (Palombo et al., 2013).

Another questionnaire, the Autobiographical Memory Characteristics Questionnaire (AMCQ) was developed to assess characteristics of autobiographical memories with 14 subscales (Boyacioglu & Akfirat, 2015). More recently, the Assessment of the Phenomenology of Autobiographical Memory (APAM) measure was created to address phenomenology of autobiographical memory with healthy adults and clinical populations with a web version for online self-administration (Vannucci et al., 2020, 2021).

These questionnaires have been used in a variety of studies examining false memories, aging, and cognitive decline (Fan et al., 2020; Mather et al., 1997; Siedlecki et al., 2015). These questionnaires share several characteristics and have generally built upon each other to fill gaps and address different aspects of autobiographical memory, which allows for targeted use depending on aims of the assessment.
